# Recognition of *Plasmodium falciparum* gametocyte surface antigens by plasma antibodies in asymptomatic Ghanaian school children

**DOI:** 10.1186/1475-2875-11-S1-P24

**Published:** 2012-10-15

**Authors:** Bismarck Dinko, Teun Bousema, Colin J Sutherland

**Affiliations:** 1 Department of Immunology and Infection, London School of Hygiene and Tropical Medicine, London, UK

## Background

Malaria transmission-reducing interventions are key components of malaria control and elimination [1]. However, little is known about the immune responses directed at circulating *Plasmodium falciparum* gametocytes in humans, knowledge of which would be useful in the development of anti-gametocyte vaccines, which would have the capability to reduce malaria transmission from humans to mosquitoes. In a study in the Gambia, mature gametocyte-infected erythrocytes of *P. falciparum* were found to carry antigens (gametocyte surface antigens, GSA) that were recognised by malaria patient’s plasma antibodies. These anti-GSA antibodies, taken at a single timepoint, were weakly associated with lower duration of gametocyte carriage in these treated patients [2,3]. We then sought to determine longitudinal patterns in GSA antibody prevalence and its relationship to possible immune suppression of gametocyte carriage *in vivo.*

## Materials and methods

Flow cytometry of cultured gametocyte-infected erythrocytes from 3D7 and from two recently adapted gametocyte-producung lines was used to detect and measure plasma antibodies recognising the erythrocyte surface. Plasma was obtained from asymptomatic *P./falciparum-positive* children attending school in a rainforest region in Ghana. These children were treated with dihydro-artemisinin piperaquine, and followed up weekly for 1 month.

## Results and conclusions

By microscopy, 8.9% (15/168) of the children enrolled carried gametocytes and a further 20% of them developed gametocytes during subsequent follow-up. (NASBA is also now being carried out to identify sub-microscopic gametocyte carriers.) Preliminary results from 113 samples tested in flow cytometry show that more than 50% of those in the sub-group of children with gametocytes at enrolment carry antibodies to GSA, and we expect this proportion to increase as gametocytes are developed during the follow-up. Further longitudinal flow cytometry, and NASBA analyses will enable us to understand the dynamics between immune responses to gametocytes and gametocyte carriage following treatment of asymptomatic malaria.
